# Relationship between the coronavirus disease 2019 pandemic and immobilization syndrome

**DOI:** 10.6061/clinics/2021/e2652

**Published:** 2021-02-01

**Authors:** Pérola Grinberg Plapler, Daniel Rubio de Souza, Helena Hideko Seguchi Kaziyama, Linamara Rizzo Battistella, Tarcisio Eloy Pessoa de Barros-Filho

**Affiliations:** I Instituto de Ortopedia e Traumatologia (IOT), Hospital das Clinicas HCFMUSP, Faculdade de Medicina, Universidade de Sao Paulo, Sao Paulo, SP, BR; II Instituto de Medicina de Reabilitacao (IMRea), Hospital das Clinicas HCFMUSP, Faculdade de Medicina, Universidade de Sao Paulo, Sao Paulo, SP, BR

## INTRODUCTION

Disuse syndrome due to immobility and/or prolonged bed rest is characterized by the deterioration of body systems secondary to musculoskeletal inactivity (1). The consequences of prolonged bed rest and lack of physical activity include effects on cognition, muscles, bones and joints, and skin, as well as the cardiovascular and respiratory systems (2). Differences in loss and atrophy among various muscles can be explained by their fiber characteristics and functions. For instance, the rectus femoris muscle, which performs primarily fast contractions, is composed mainly of type II fibers. In contrast, the vastus intermedius is a slow-twitch muscle comprising mainly of type I fibers. The rate of loss of muscle tropism is faster in the rectus femoris muscle (9%) than that in the vastus intermediate (1%) (3).

Contractures that commonly appear due to immobility limit the range of motion and may impede the normal performance of their function. Prolonged joint immobility is the most significant risk factor for arthrofibrosis. Greater loss has been reported in the lower limbs compared to that in the upper limbs. Malnutrition and lack of mobility lead to rapid loss of bone mass due to lack of muscle activity, lack of weight bearing, low calcium intake, and lack of sun exposure (4).

Critical illness is related to a state of catabolic stress, in which patients commonly present a systemic inflammatory response, which is associated with complications that promote multiple organ dysfunction, prolonged hospitalization, and increased morbidity and mortality (5,6).

In severe disease, skeletal muscle consumption occurs due to the body’s inability to maintain a balance between protein synthesis and degradation (7,8). Increased muscle protein degradation occurs via intracellular signaling pathways (9). Among them, the ubiquitin-proteasome system is the main pathway related to the proteolysis mechanism, in which two specific enzymes related to skeletal muscle atrophy are activated in response to inactivity and the inflammatory process (6,10).

The effects of prolonged immobility include muscle myopathy and atrophy, resulting in musculoskeletal deconditioning. Patients with prolonged immobility can lose up to 2% of their lean body mass per day, resulting in poor balance and coordination, along with joint stiffness (11,12).

Before the 1990s, little attention was given to post-intensive care unit (ICU) procedures and long-term follow-up studies were rare; however, the side effects and sequelae resulting from prolonged ICU stay have recently received increased attention (11).

Prolonged immobility is not uncommon in the ICU and can result in joint limitations and, more rarely, peripheral nerve injuries, as well as impaired mechanical ventilation. Additionally, nutritional deficits may occur due to the difficulty or impossibility of ingestion and, depending on the length of stay, may have other clinical consequences. Regardless of the profile of the hospitalized patient, efforts must be made to understand their characteristics and the possible consequences of hospitalization, which requires the integration of clinical care and rehabilitation (11).

Seriously ill patients in the ICU are subjected to different drug treatments and long periods of immobility; moreover, bed rest may result in a worsening of global muscle weakness termed “ICU-acquired muscle weakness” (6).

Muscle weakness is widespread and frequent in patients who are in the recovery phase after severe illness. This decrease in strength may be accompanied by both muscle and joint damage. Although the definitive reason for these symptoms remains unknown, their origin is attributed to increased catabolism, leading to protein degradation and low levels of muscle formation or regeneration (13).

Hospitalization time after intensive care also generates high financial burdens (14). Loss of muscle strength is common in healthy individuals after prolonged physical inactivity or, in immobilization syndrome, due to prolonged bedridden status (6). The loss of skeletal muscle mass in critically ill patients may also be related to the metabolic changes most commonly seen in these patients (hypermetabolism and hypercatabolism) associated with immobilization and lack of adequate nutritional support (6,15,16).

Even patients without previous neuromuscular disease have muscle limitations owing to prolonged immobilization. These limitations are generally symmetrical, bilateral, and pronounced (16). The net reduction of the myosin/actin ratio after an ICU stay is referred to as critical illness myopathy. The more fragile people were before admission and the longer they remained on mechanical ventilation, the worse the consequences. The main sequelae include joint stiffness, which followed long immobilization, and a fear of falling owing to a lack of balance due to musculoskeletal changes. These effects make it difficult to use stairs, even 8 weeks after discharge, according to a study in patients with long-lasting ICU stays (17). Mobility in outdoor activities was also compromised in those who remained on mechanical ventilation for longer periods (6,18). The numerous interacting factors include advanced age, disease severity, and the treatment itself, which cause a loss of muscle mass and consequent impairment of muscle function (19). The complications of this deconditioning can persist for up to 12 months after discharge (11,20,21).

Patients with coronavirus disease 2019 (COVID-19) may have skeletal muscle consequences caused by long ICU stays with immobilization in the prone position. These problems include severe muscle weakness and fatigue, joint stiffness, dysphagia, (neuro)psychological problems, and impaired functioning affecting mobility, daily living, and work activities (22).

As COVID-19 is a new disease, much has been deduced from previous studies on patients with other long-term illnesses. Early intense physical exercise or activities are not well tolerated, with rapid oxygen desaturation. The reported issues include muscle weakness, critical illness myopathy and neuropathy, dysphagia, reduced joint mobility, neck and shoulder pain (due to a prolonged prone position), difficulty in standing, changes in balance and gait, limitations in activities of daily living (ADL), changes in memory, mental confusion, and emotional problems. These impairments occurring during the post-acute phase must be the targets of the rehabilitation process in medium-and long-term monitoring (22-24).

Among 43 papers identified in a search of the PubMed database on June 7, 2020, using the terms “covid 19” and “muscle,” few reported muscle pain as one of the symptoms of COVID-19 and only one (22), cited immobilization syndrome, with decreased muscle strength (Table 1).

Muscle function is impaired early during ICU stay. After 7 days of bed rest, peripheral muscle strength may decrease by approximately 20%, with additional losses of 20% of the remaining strength for each subsequent week owing to the inflammatory condition that reduces muscle protein synthesis and increases its degradation (1,6,19).

Muscle mass is intrinsically linked to the muscle's ability to generate strength. Gruther et al. (19) observed a greater loss of muscle mass between the second and third weeks of bed rest, while Puthucheary et al. (33) observed greater muscle loss in the first week of ICU admission, a fact that can be explained by the increased protein degradation observed on the first day of admission to the ICU, to the detriment of muscle protein synthesis (6).

Striated skeletal muscle is generally formed by the organization of actin and myosin filaments and is necessary for the generation of muscle strength. In acquired muscle weakness, loss of myosin filaments is observed, associated with the rupture of actin filaments (8). Type II muscle fibers become more sensitive to the inflammatory process, which occurs in the critical phase of severe disease, becoming more susceptible to atrophy resulting from muscle disuse, a condition more evident in fast-twitch fibers (6,8).

The notion that the treatment of critical illness ends at ICU discharge is not entirely accurate. Most adults treated in the ICU survive their critical illness, producing an expanding group of survivors that may have serious morbidities as the aftereffects of both the critical illness and its treatment. These morbidities have a substantial impact on the patients as well as their families and society (14,34,35).

Specifically, many ICU survivors develop post-intensive care syndrome (PICS) (24,36), which includes physical, psychological, and cognitive impairments (37,38). PICS can persist for years after a patient leaves the ICU, adversely affecting patients and their families (14,39,40).

The goal is to prevent sequelae and ensure rehabilitation during hospitalization and after discharge. As many of the sequelae are related to the locomotor system, exercising is a mandatory part of treatment after discharge. Concerning disability, physical activities should be scheduled to slowly and progressively increase in intensity until the return to pre-hospitalization capacity. A randomized study demonstrated the effectiveness of a 6-week rehabilitation program aimed at self-help in reducing depression and improving physical capacity compared to less innovative treatments (11,41).

However, with the severity of the pandemic, efforts were aimed at the survival of the possible largest number of patients, and rehabilitation-focused care was neglected.

Early rehabilitation to prevent loss of function can positively affect functionality awareness and self-care ability. It makes all the differences in the long run, with faster gain in physical and emotional independence, lower rates of readmission, and fewer deaths (14).

In conditions that require long ICU stays with the patient immobilized in bed, such as on a respirator for an average of 15 days in a prone position, the consequences can last for even longer periods. These include extreme fatigue and muscle weakness, joint stiffness, dysphagia, neuropsychological changes, mobility impairment, and limitations in exercise, ADL, and work activities (22).

A necropsy study of patients with COVID-19 collected tissue samples under ultrasound guidance from the lungs, liver, kidneys, spleen, and heart, while other tissues were sampled without direct image guidance from skeletal muscle, skin, and brain. The authors observed systemic inflammation or shock, myositis, and other consequences such as myocarditis, endothelial changes in small vessels, and reactive gliosis in the brain. Moreover, patients who required prolonged prone positioning during mechanical ventilation experienced posterior reversible encephalopathy syndrome and critical illness myopathy/neuropathy following acute respiratory distress syndrome. Some patients also showed contractures in plantar flexion, shortening of tendons, and bedsores resembling neuromuscular diseases. As these patients may have severe respiratory impairment and may not tolerate intense exercise, they require comprehensive rehabilitation care (42).

Reports of prolonged ICU stay among patients with COVID-19 reinforce the need for rehabilitation care in and between treatment phases.

## AUTHOR CONTRIBUTIONS

Plapler PG was responsible for the literature review and the first draft of the manuscript. Souza RB and Kaziyama HHS contributed equally to the manuscript writing. Battistella LR and Barros-Filho TEP contributed equally to the manuscript supervision, general structure review and manuscript writing.

## Figures and Tables

**Table 1:** Papers published in PubMed as of June 7, 2020, that cited muscles and the percentage of symptoms (muscle soreness, fatigue, or myalgia), in patients with COVID-19.

Muscle ache (11%)	DOI: 10.1016/S0140-6736(20)30211-7 - Epidemiological and clinical characteristics of 99 cases of 2019 novel coronavirus pneumonia in Wuhan, China: a descriptive study (25)
Muscle soreness (33%)	DOI: 10.1002/jmv.25884 - Clinical characteristics of 3,062 COVID-19 patients: a meta-analysis (26)
Muscle soreness or fatigue (35.5%)	DOI: 10.1002/jmv.25822 - Imaging and clinical features of patients with 2019 novel coronavirus SARS-CoV-2: a systematic review and meta-analysis (27)
Myalgia or fatigue (35.8%)	DOI: 10.1002/jmv.25757 - COVID-19 patients' clinical characteristics, discharge rate, and fatality rate of meta-analysis (28)
Muscle ache (among other symptoms) at admission as predictive factors for the severe/critical subtype	DOI: 10.1016/j.ijid.2020.03.040 - Epidemiological, clinical characteristics of cases of SARS-CoV-2 infection with abnormal imaging findings (29)
Myalgia or fatigue (52%)	DOI: 10.1136/bmj.m792 - Clinical findings in a group of patients infected with the 2019 novel coronavirus (SARS-Cov-2) outside of Wuhan, China: retrospective case series (30)
Fatigue or myalgia (70%)	DOI: 10.3760/cma.j.issn.1001-0939.2020.03.014 - Clinical characteristics of 30 medical workers infected with new coronavirus pneumonia (31)
Cachexia and sarcopenia	DOI: 10.1002/jcsm.12589 - COVID‐19: a major cause of cachexia and sarcopenia? (32)
